# Endoscopic-Assisted Transoral Approach (EATA) for Extracranial Tumors: A Multicentric Case Series

**DOI:** 10.3390/life15060975

**Published:** 2025-06-18

**Authors:** Giovanni Motta, Arianna Di Stadio, Luca D’Ascanio, Luigi D’avino, Vincenzo della Peruta, Francesco Chiari, Carlo Magno, Giuseppe Tortoriello, Gaetano Motta

**Affiliations:** 1 Otolaryngology Head and Neck Surgery Unit, “Azienda Ospedaliera di Rilievo Nazionale dei Colli, Ospedale Monaldi”, 80131 Naples, Italy; giovannimotta95@yahoo.com (G.M.); orldavino@gmail.com (L.D.); vincenzodellaperuta@gmail.com (V.d.P.); dott.giuseppetortoriello@virgilio.it (G.T.); 2 ENT Unit, Department of Mental, Physical Health and Preventive Medicine, University of Campania “Luigi Vanvitelli”, 80131 Naples, Italy; gae.motta@libero.it; 3 Otorhinolaryngology Head and Neck Surgery, Azienda Ospedaliera “Ospedale Riuniti Marche Nord”, 61122 Pesaro, Italy; l.dascanio@gmail.com; 4 Otolaryngology, Head and Neck Unit, “Santo Spirito” Hospital, 65124 Pescara, Italy; francesco.chiari.med@gmail.com; 5 Clinica Vesuvio, 80147 Naples, Italy; carlomagno@inwind.it

**Keywords:** Endoscopic Assisted Transoral Approach (EATA), transoral robotic surgery (TORS), benign tumors, pleomorphic adenoma, schwannoma, parapharyngeal space (PPS), masticator space (MS)

## Abstract

Endoscopic surgery is useful and helpful especially to access areas with limited visibility. The combination of this technique with innovative approaches could be the solution to improve quality of surgery and patients’ outcome. This study aimed to illustrate how Endoscopic-Assisted Transoral Approach (EATA) can be used to successfully remove specific extracranial tumors with defined characteristics. Eleven patients with extracranial tumors underwent surgical resection using an EATA between 2003 and 2025. All patients underwent clinical examination and fiberoptic laryngoscopy. Preoperative CT and/or MRI was performed in all cases. All patients were successfully treated utilizing an EATA. Histological examination revealed nine parapharyngiomas, comprising five pleomorphic adenomas, two schwannomas, one ectopic thyroid gland, one lipoma, one masticator space schwannoma, and one nasopharyngeal pleomorphic adenoma. No intra-operative nor peri-operatory complications were observed. The only long-term sequela observed was Horner’s syndrome in the two schwannomas originating from the parapharyngeal-carotid space. The mean hospital stay was 2.6 days, while the mean follow-up duration was 9.8 years. The EATA represents a valid surgical technique for the treatment of benign, encapsulated, and non-vascular parapharyngeal space (PPS) tumors exhibiting posterior displacement of major vessels. This approach may also prove beneficial for the management of other benign, encapsulated, and non-vascular tumors located in the nasopharynx and masticator space.

## 1. Introduction

Within the extensive spectrum of expansive lesions affecting the head and neck region, certain extracranial tumors, exhibiting specific characteristics and involving particular spaces of the cervicofacial district, are generating considerable discussion [1,2,3,4,5,6,7,8,9]. The optimal surgical approach for these lesions—whether open external approaches, transoral robotic surgery (TORS), conventional transoral surgery, or Endoscopic-Assisted Transoral Approaches (EATA)—remains a subject of debate, particularly concerning tumors arising in the PPS, occasionally the masticator space, the nasopharynx, and the oropharynx [1,2,3,4,5,6,7,8,9]. These anatomical regions, where head and neck surgeons operate via open, endoscopic transoral, and robotic transoral routes, can be affected by neoplasms of different nature, both benign and malignant [1,2,3,4,5,6,7,8,9]. In light of the technological and scientific advancements of recent years, the optimal surgical strategy to manage this broad range of pathologies has become a topic of discussion [1,2,3,4,5,6,7,8,9].

In the realm of malignant oncological disease, especially within regions like the oropharynx, transoral robotic surgery is gaining significant traction [2,3,4]. Transoral approaches, both endoscopic and robotic, are also the subject of important debates in managing the pathology of difficult-to-access spaces such as the PPS, where open transcervical or transcervical–transparotid approaches have historically been the mainstay. Transoral approaches to pathology in these spaces, including the PPS, have been criticized in the past literature primarily due to the inadequate exposure of the approach, which restricts tumor visualization, leading to an increased risk of neurovascular injury and a higher risk of tumor spillage and recurrence over time [1,5,7].

However, in recent years, various authors have reported interesting case series utilizing both robotic and endoscopically assisted transoral techniques, demonstrating promising outcomes when the pathology is appropriately selected (i.e., only tumors with specific characteristics) [1,5,6,7]. In particular, the EATA is being re-evaluated for certain pathologies in these districts, with several authors emphasizing its efficacy and safety when carefully selecting appropriate cases based on specific tumor characteristics [1,6,7].

The endoscope guarantees a full direct vision of the anatomy even in minimal space, thanks to this characteristic the combination of endoscope with traditional technique as, for example, retro-sigmoid approaches to the skull base has allowed for minimizing the risk of this surgery and optimizing the outcome [10]. The use of the endoscope has been recently proposed even for performing procedure in clinical setting thanks to the benefit reachable with this technique [11].

Based on the previous studies, we think that the endoscope could be useful to improve the outcomes of transoral surgery, minimize the risk related to this minimal access and take advantages from this approach.

In this multicentric retrospective case series, we described our experience with EATA, and the considerations regarding the feasibility of an EATA used for tumors in different areas of the head district. We also detailed the types of tumors amenable to this technique and their defining characteristics.

## 2. Materials and Methods

This retrospective, multicentric case series included eleven consecutive patients with extracranial head and neck tumors who underwent surgical resection via an EATA between January 2003 and April 2025 at three specialized Italian head and neck centers. The study cohort comprised nine PPS tumors, with seven specifically originating from the true PPS and two from the carotid space portion of the PPS. The remaining two cases included one schwannoma of the MS and one pleomorphic adenoma of the nasopharynx. The 11 lesions underwent surgical management by three distinct surgeons. The 9 parapharyngeal tumors were operated on at the Otorhinolaryngology Unit of Vanvitelli University by the paper’s senior author (G.M.). Furthermore, co-author L.D (Luca D’Ascanio) performed the surgery for the masticator space schwannoma at the ‘Ospedale Riuniti Marche Nord’ Hospital, and co-author C.M. carried out the resection of the nasopharyngeal pleomorphic adenoma at Clinica Vesuvio.

Patient data were retrospectively collected from institutional electronic medical records. Preoperative evaluation for all patients included a comprehensive clinical examination and fiberoptic laryngoscopy to assess the tumor’s extent and relationship to surrounding structures. Preoperative imaging, consisting of computed tomography (CT) and/or magnetic resonance imaging (MRI), was performed in all cases to delineate tumor size, location, and the involvement of adjacent anatomical spaces.

Surgical procedures were performed using an endoscopically assisted transoral technique. Given that the lesions were treated across multiple centers and by different surgeons, and involved different anatomical sites, the specific surgical procedure varied. This variation was contingent upon the characteristics and location of the lesion, as well the individual practices and preferences of the surgeons involved. Speaking generally, the procedure had common characteristics that were approached through the mouth, using the endoscope to identify (i) the characteristics of the mass (capsulated, not-capsulated, color, presence of superficial vessels, and site of vascular peduncle), (ii) relationship with surrounding tissue, (iii) identification of bleeding, and (iv) double-check of the operatory site for controlling cauterization and the complete removal of the mass. In general, our study focused on the feasibility of the EATA for selected lesions amenable to this technique. Hence, the specific surgical approach and instrumentation varied based on tumor location and size, as determined by the operating surgeon.

Postoperative data collected included the duration of hospital stay (in days) and any immediate or long-term postoperative sequelae. Recurrence was defined as clinical or radiologic evidence of tumor regrowth at the surgical site.

Data analysis included descriptive statistics to summarize patient demographics, tumor characteristics, surgical outcomes, and follow-up data.

## 3. Results

Eleven patients with extracranial head and neck tumors underwent EATA resection between 2003 and 2025. The cohort included seven true PPS tumors, two carotid space PPS tumors, one MS schwannoma, and one nasopharyngeal pleomorphic adenoma (NPA).

The full endoscope-assisted procedure was performed in one case (MS schwannoma) while in the other 10 cases the endoscope was used “in” and “out” to perform the passages described in the Material and Methods Section.

**Figure 1:**
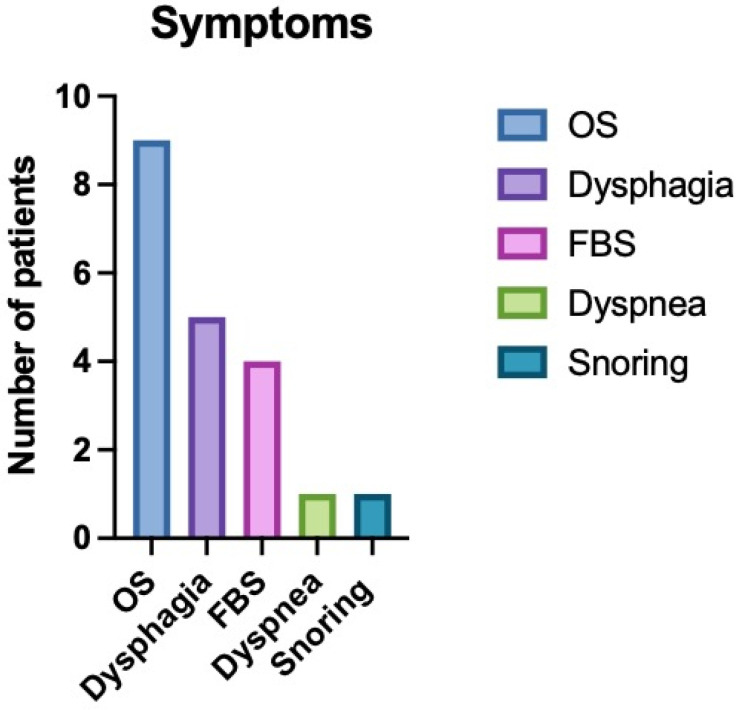
The graph describes the symptoms more commonly referred by the patients. OS: perception of oropharyngeal mass; FBS: foreign body sensation. Some patients presented more than a symptom at the same time; in particular, OS was commonly associated with dysphagia.

The median age of the patients was 56 years (range: 36–86 years), with a female predominance (81.8%) The most common presenting signs and symptoms were related to an oropharyngeal mass (nine patients), including dysphagia (five patients), foreign body sensation (four patients), dyspnea (one patient), and snoring (one patient) (Figure 1). Two patients presented asymptomatically (one with a true PPS lipoma and one with a MS schwannoma), while one patient with a nasopharyngeal pleomorphic adenoma presented with bilateral nasal obstruction, snoring, and bilateral otitis media with effusion.

**Figure 2:**
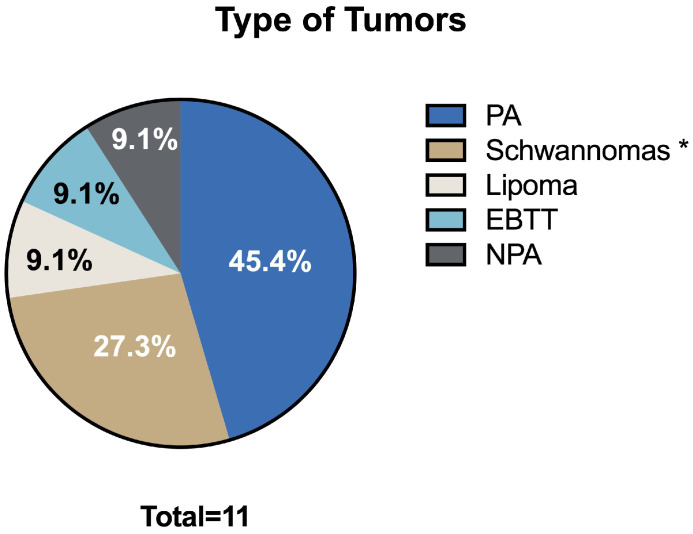
Mostly of the patients described in this case series were affected by pleomorphic adenoma (PA), followed by schwannoma (*: including one in the masticatory space (MA). Lipoma, ectopic benign thyroid tumor (EBTT), and nasopharyngeal pleomorphic adenoma (NPA) were rarely observed).

The median surgery time was 50 min (range: 10–120 min). No intraoperative complications were reported across the entire cohort. The median tumor size was 3.7 cm in the largest dimension (range: 1.9–6 cm). Histological analysis revealed a variety of benign tumors: five pleomorphic adenomas, two schwannomas, one lipoma, one ectopic benign thyroid tissue, one MS schwannoma, and one nasopharyngeal pleomorphic adenoma (Figure 2).

The median hospitalization time was 2.6 days (range: 2–5 days). Postoperative sequelae were observed in two patients (18.2%), both of whom experienced Horner’s syndrome following resection of schwannomas originating from the carotid space.

The median follow-up period was 9 years (range: 1–21 years). No cases of tumor recurrence were observed during the follow-up period in this series. These results are summarized in Table 1 (resumptive table).

## 4. Discussion

In the realm of head and neck tumor pathology, contemporary head and neck surgeons have access to many surgical strategies to address patient needs. Alongside traditional open approaches, recent technical and scientific advancements have paved the way for novel surgical approaches and innovative techniques, such as TORS and endoscopically assisted transoral surgery. The selection of the surgical approach and technique is contingent upon the specific tumor pathology and the anatomical location of the lesion affecting the patient. Indeed, head and neck pathology exhibits significant heterogeneity, both in terms of the histological diversity and the numerous sites where various neoplasms can arise [1,2,3,4,5,6,7,8,9].

Transoral approaches, whether robotic or endoscopically assisted, have witnessed an increasing adoption in recent years for the treatment of both benign and malignant conditions [1,2,3,4,5,6,7,8,9]. Analyzing various cervicofacial regions, these techniques have been extensively utilized. Notably, several studies have highlighted the significant and potentially expanding role of robotic surgery in the management of oropharyngeal carcinomas and, in select cases, supraglottic laryngeal carcinomas [2,3,4]. Conversely, in the context of malignant disease, endoscopically assisted transoral surgery appears to hold promise as a salvage surgery option for recurrent nasopharyngeal carcinomas [8,9].

**Figure 3:**
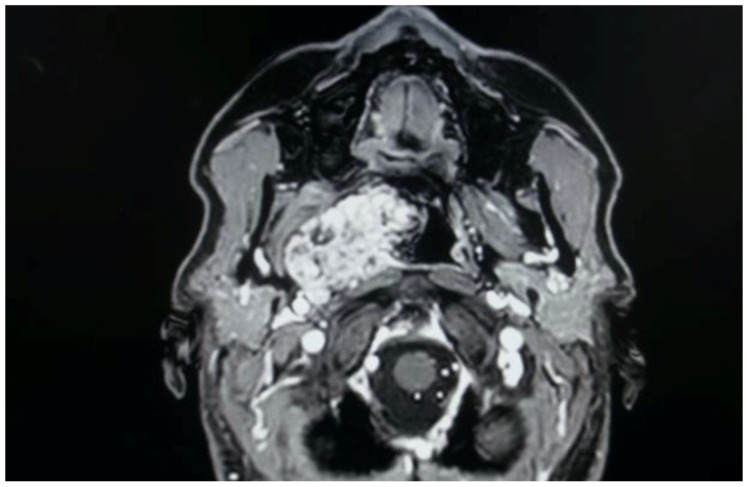
Pleomorphic adenoma originating from the true PPS with posterior major vessels’ dislocation.

The treatment of PPS pathology represents a territory where both transoral approaches have been proposed, even as alternatives to classic open transcervical or transcervical–transparotid approaches [1,5]. Numerous authors in recent years have described the safety and efficacy of these transoral techniques, demonstrating favorable outcomes in terms of postoperative pain, length of hospital stay, and aesthetic results compared to traditional external approaches [1,5]. Our presented case series, as highlighted in the summary table, corroborates these findings. Specifically, in our cohort, postoperative pain was never significant (managed with as-needed paracetamol when required), hospitalization was brief, and no patient necessitated an external incision, offering advantages in terms of both postoperative recovery and aesthetic outcomes. This shift towards the selection of a transoral corridor is gaining momentum; however, it is crucial to emphasize several aspects. The meticulous evaluation of the patient and the tumor is paramount when considering a transoral approach [1,5,6,7]. A thorough clinical and radiological assessment is essential, particularly to evaluate the benign or malignant characteristics of the lesion, its vascularity in the case of vascular lesions, and its relationship with major neck vessels, which could lead to critically challenging management issues if these evaluations are not diligently performed [1,5,6,7]. Many case series highlight that tumors removed via these approaches are predominantly benign, non-vascular, and encapsulated lesions, such as pleomorphic adenomas and schwannomas. Malignant tumors, where open approaches are often more appropriate, and vascular tumors like paragangliomas, due to the high risk of intraoperative hemorrhage and subsequent difficult management via a transoral route, are typically excluded from these techniques [1,5]. Consequently, magnetic resonance imaging (MRI) with contrast enhancement frequently serves as the primary imaging modality to confirm clinical assessment and guide the decision-making process for selecting suitable candidates for transoral surgery [1,5]. In this presented multicentric case series, all benign and encapsulated lesions removed via the transoral route exhibited a favorable pattern of major vessel displacement. Specifically, the seven true PPS tumors, comprising five pleomorphic adenomas originating from ectopic salivary tissue, minor salivary glands, and the deep lobe of the parotid gland, consistently demonstrated a posterior displacement of the major neck vessels and an anteromedial displacement pattern of the parapharyngeal fat, a characteristic typically associated with benign lesions of the true PPS [1,5]. Figure 3 illustrates a pleomorphic adenoma from our case series, originating from accessory salivary glands of the true PPS, demonstrating this characteristic pattern of posterior displacement.

**Figure 4:**
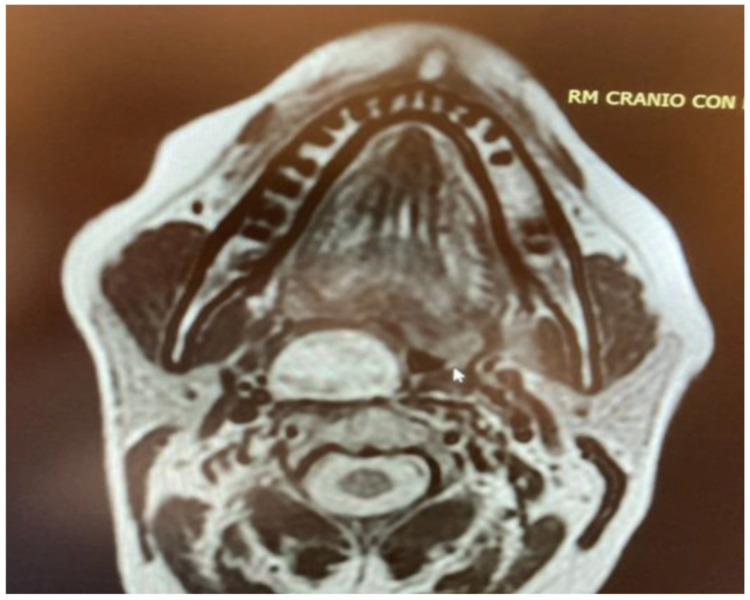
PPS schwannoma showing atypical posterior major vessels displacement.

The other two true PPS lesions, an ectopic thyroid gland and a lipoma, also presented with a favorable displacement pattern, with the major vessels situated posteriorly to the lesions. Regarding the two remaining parapharyngiomas, which were schwannomas of the cervical sympathetic chain, the transoral approach was feasible only due to an atypical displacement pattern. Despite originating from the carotid space, the major vessels exhibited a posterior displacement rather than the anterior displacement typically expected with lesions of the carotid space (Figure 4). Consideration should be given to the preservation of neural and vascular structures within the PPS. It is imperative to safeguard major vascular structures, such as the internal carotid artery and internal jugular vein, particularly since injury to the internal carotid artery during a transoral approach could lead to catastrophic outcomes. Indeed, in our case series, no damage to these major vessels has ever occurred. However, regarding neurogenic lesions originating from the carotid space, a distinct consideration applies to schwannomas. As these tumors originate from nerves, even if the nerve of origin can be preserved during resection, this does not guarantee the preservation of its function [1,12,13,14]. In our presented case series, for instance, two cases of schwannoma originating from the sympathetic chain resulted in Horner’s syndrome. Therefore, accurate radiological diagnosis of PPS lesions, especially those within the carotid space, is crucial for guiding the surgeon regarding the nerve of origin. Nevertheless, it must be highlighted that schwannomas do not always exhibit a typical pattern of vascular or neural displacement, and our case series serves as an illustrative example of this variability.

**Figure 5:**
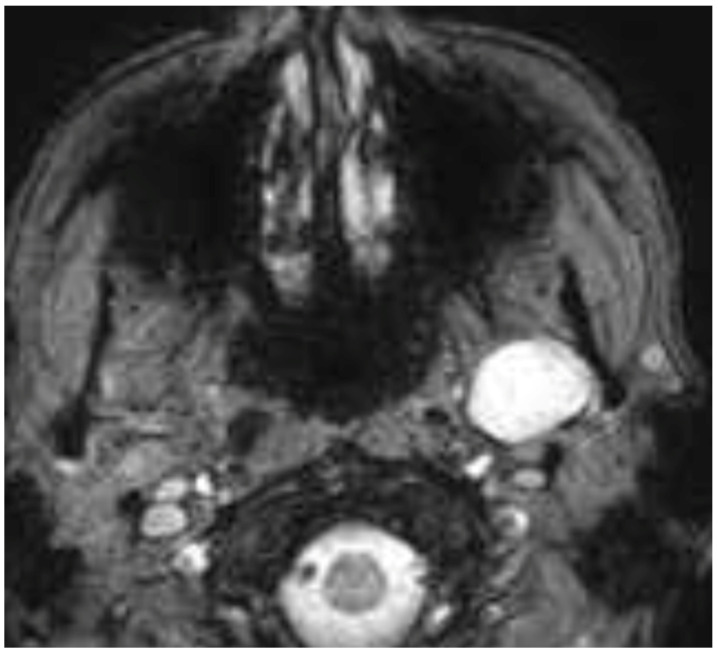
Masticator space schwannoma.

**Figure 6:**
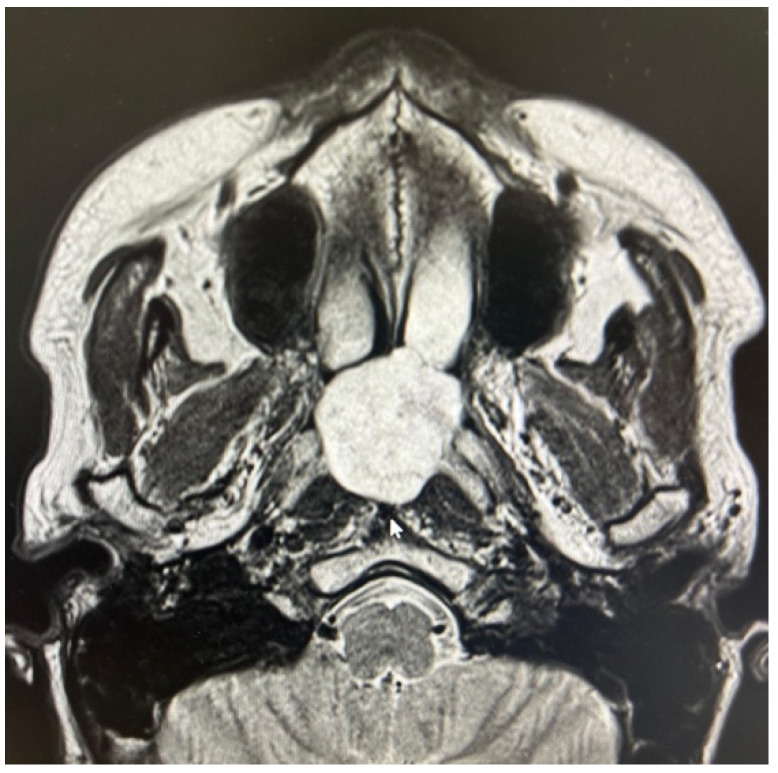
Pleomorphic adenoma of the nasopharynx.

For the final two lesions, which did not arise within the PPS, the issue of favorable major vessel displacement was not a primary concern. Both the schwannoma of the MS (Figure 5) and the NPA (Figure 6) were located outside the immediate vicinity of major vessels posing a positional challenge. Therefore, the transoral approach was feasible due to their favorable location and benign characteristics, both clinically and radiologically confirmed by MRI.

What remains to be investigated in the literature is a direct comparison between TORS and endoscopically assisted transoral surgery for the treatment of these lesions, as the available data to compare the safety and efficacy of the two techniques are still limited. While the diagnostic work-up for selecting lesions amenable to a transoral approach remains consistently the same regardless of the subsequent surgical modality (robotic or endoscopic) [1,5,12,13,14,15], certain considerations currently favor the EATA, as utilized in this multicentric case series. An EATA is feasible across a wider range of healthcare settings and allows for a more dynamic surgical intervention, providing tactile feedback of the lesion—a feature absent in the otherwise highly precise robotic dissection. The absence of tactile feedback may also contribute to the potential for capsule disruption during robotic dissection [12,13,14,15]. Therefore, while robotic dissections appear safe and effective, certain limitations exist, such as the potential for capsular rupture during the robotic procedure. Consequently, these patients require further investigation and long-term follow-up to accurately assess recurrence rates, particularly for lesions like pleomorphic adenomas. On the other hand, surgical times associated with the EATA are notably shorter when compared to TORS. As evidence of this, our case series, although limited, has confirmed that surgical times are shorter compared to robotic dissections [5,12,13]. More importantly, no recurrences have been observed in the removed lesions, particularly in the six pleomorphic adenomas excised using this technique. In addition, there are undeniable advantages in terms of cost effectiveness and the broader availability of endoscopic equipment compared to robotic systems, which are not universally accessible [1,5,12,13,14,15].

Future studies are warranted to clarify these aspects, directly comparing the two transoral techniques and elucidating their respective advantages and disadvantages.

**Figure 7:**
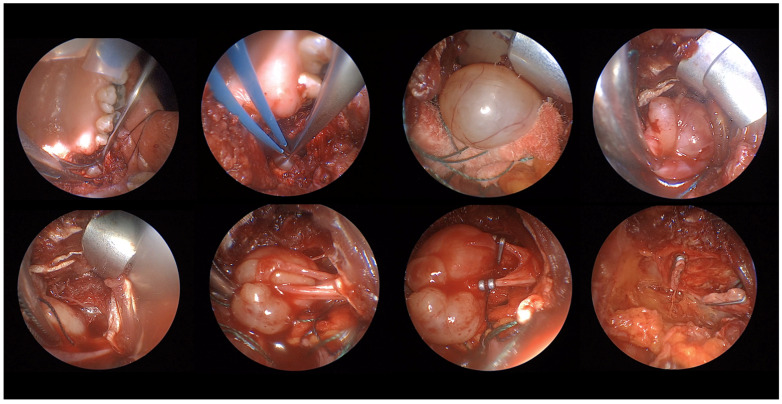
The figure shows all the advantages of using an endoscope for transoral approach. From up-left to down-right the endoscope allows to identify the mass, to immediately cauterize small vessels to maintain clean the area, the full visualization of finding and its capsule and its posterior relationship. Moreover, the clear visualization of the vascular structures of the tumor allows an easy clamping with consequent excellent bleeding control at the end of the surgery.

Overall, based on what we observed in this case series, EATA presents several advantages. Firstly, it is an excellent view of the operatory area and allows for clear identification of the mass and surrounding structures, easy identification of the vessels and their clamping, excellent control of the bleeding, and easy evaluation of the result post-mass removal (Figure 7). What emerges from this case series is that lesions amenable to a transoral corridor, potentially with endoscopic assistance, include encapsulated tumors of the true PPS that present with oropharyngeal bulging, anteromedial medialization of parapharyngeal fat, and posterior displacement of major vessels (primarily pleomorphic adenomas); encapsulated benign tumors of the carotid PPS that present with oropharyngeal bulging but exhibit an atypical vascular displacement pattern (i.e., posterior displacement of major vessels, despite being carotid space lesions), a class primarily comprising schwannomas; encapsulated tumors of the masticator space, even if they do not present with intraoral bulging; and encapsulated lesions protruding into the rhino-oropharyngeal lumen where major vessel displacement is not a concern. Magnetic Resonance Imaging (MRI) with a contrast agent plays a crucial role in the differential diagnosis of these spaces and in defining the characteristics of the lesions in question, in addition to its ability to exclude vascular lesions by highlighting flow voids.

### Limitations of the Study

This study presents some limitation as first two of these pleomorphic adenomas have a follow-up period of less than three years, and therefore, it is not yet possible to definitively rule out long-term recurrences. As second, this is a case series, and the results can change in larger sample. Then for some surgeons, i.e., maxillo-facial, combining transoral approach with an endoscope could be difficult and time consuming; the surgeons who performed this surgery were all otolaryngologists. In ENT, the use of endoscope is very common (nasal surgery, cranial base, etc.) so it might be easy adopting this type of technology for different approaches including transoral.

## 5. Conclusions

This multicentric retrospective case series suggests that the endoscopically assisted transoral approach is a feasible and potentially effective technique for the removal of benign, encapsulated lesions located within the PPS, and in selected cases, of the oro-rhinopharyngeal cavity and MS. Careful clinical evaluation, corroborated and confirmed by contrast-enhanced MRI, is crucial, particularly for assessing the spatial relationship of the lesion with major vessels. Therefore, meticulous patient selection is paramount in identifying suitable candidates for endoscopically assisted transoral surgery in these complex anatomical regions.

## Figures and Tables

**Table 1:** A summary of the cases included in this case series.

		Signs/Symptoms	Location	Surgery Time	Intraoperative Complications	Tumor Size (cm)	Histological Diagnosis	Hospitalization Days	Postoperative Sequalae	Follow-Up (Years)	Recurrence
86	Male	Oropharyngeal mass, Dyspnea, dysphagia	True PPS	60 min	None	4 × 3 × 5.2	Benign thyroid tissue	3	None	21	No
75	Female	Oropharyngeal mass, Dysphagia	Carotid space	70 min	None	3 × 4 × 3	Schwannoma	3	Horner Syndrome	16	No
53	Male	Oropharyngeal mass, Asymptomatic	True PPS	20 min	None	4 × 2.5 × 3	Lipoma	2	None	12	No
56	Female	Oropharyngeal mass, Dysphagia, Foreign body sensation	True PPS	40 min	None	5 × 3.3 × 4.2	Pleomorphic Adenoma	2	None	11	No
59	Female	Oropharyngeal mass, Dysphagia, Foreign body sensation	True PPS	50 min	None	5 × 3 × 3.3	Pleomorphic Adenoma	2	None	9	No
57	Female	Oropharyngeal mass, snoring, Dysphagia, Foreign body sensation	Carotid space	120 min	None	2.6 × 2.6 × 2.2	Schwannoma	3	Horner Syndrome	6	No
49	Female	Oropharyngeal mass, Dysphagia, Dysphonia, Foreign body sensation	True PPS	50 min	None	6 × 3.4 × 4.5	Pleomorphic Adenoma	3	None	3	No
55	Female	Oropharyngeal mass, Foreign body sensation	True PPS	40 min	None	4 × 2 × 2	Pleomorphic Adenoma	2	None	18	No
36	Female	Oropharyngeal mass, Asymptomatic	True PPS	30 min	None	3 × 2 × 2.2	Pleomorphic Adenoma	4	None	10	No
42	Female	Asymptomatic	Masticator Space	80 min	None	1.9 × 2.3 × 1.9	Schwannoma	5	None	1	No
49	Female	Nasal obstruction (bilateral), Snoring, Bilateral otitis media with effusion	Nasopharynx	10 min	None	2.7 × 1.5 × 2	Pleomorphic Adenoma	2	None	1	No

## Data Availability

Data are available upon reasonable request to the corresponding authors.

## Abbreviations

EATA	Endoscopic-Assisted Transoral Approach
TORS	Transoral Robotic Surgery
PPS	Parapharyngeal Space
MS	Masticatory Space
CT	Computer Tomography
MRI	Magnetic Resonance Imaging
